# Clinical Impact of Vancomycin Treatment in Ampicillin-Susceptible Enterococci Bloodstream Infections

**DOI:** 10.3390/antibiotics11121698

**Published:** 2022-11-25

**Authors:** Jatapat Hemapanpairoa, Dhitiwat Changpradub, Wichai Santimaleeworagun

**Affiliations:** 1 Department of Pharmacy Practice and Pharmaceutical Care, Faculty of Pharmaceutical Sciences, Burapha University, Chonburi 20131, Thailand; 2 Division of Infectious Disease, Department of Medicine, Phramongkutklao Hospital, Bangkok 10400, Thailand; 3 Department of Pharmaceutical Care, Faculty of Pharmacy, Silpakorn University, Nakorn Pathom 73000, Thailand; 4 Pharmaceutical Initiative for Resistant Bacteria and Infectious Diseases Working Group, Faculty of Pharmacy, Silpakorn University, Nakorn Pathom 73000, Thailand

**Keywords:** vancomycin, ampicillin, enterococcus, bacteremia, ampicillin-sensitive

## Abstract

Enterococci are major causes of bacteremia. Although the mortality rate of ampicillin- susceptible enterococci (ASE) bloodstream infections (BSI) is lower, compared with that of ampicillin-resistant enterococci BSI, the role of treatment regimens in ASE BSI remains to be determined. This retrospective study aimed to evaluate the treatment outcomes and factors associated with mortality among patients with ASE BSI. The charts of 145 enrolled patients with ASE BSI between January 2013 and April 2022 at Phramongkutklao Hospital were reviewed. The 30-day and in-hospital mortality rates were 28.8 and 41.9%, respectively. The 30-day mortality rate was higher in the vancomycin treatment group than in the beta-lactam treatment group (61.5 vs. 26%; *p* = 0.02). Pitt bacteremia score (OR 1.44, 95% CI 1.20–1.71); age-adjusted Charlson Comorbidity Index (OR 1.34, 95% CI 1.14–1.58); and vancomycin treatment (OR 4.07, 95% CI 1.02–16.22) were independent factors associated with 30-day mortality. The severity of illness, comorbidity and definitive therapy with vancomycin increased the mortality rate of patients with ASE BSI. Anti-enterococcal beta-lactams remain the first line antibiotics for ASE bacteremia.

## 1. Introduction

Enterococci are gram-positive bacteria that are commensal in the gastrointestinal tract and are common pathogens in the community and hospitals. Enterococci are major causes of bacteremia, endocarditis, intraabdominal infection, pelvic infection, urinary tract infection and intravascular infection. *Enterococcus faecalis* and *Enterococcus faecium* are the common species that cause enterococcal infection. They can bring about major medical problems and are difficult to treat, because they can survive in the environment, are resistant to several antibiotics, and are tolerant to cell wall-active agents, e.g., beta-lactams and vancomycin, that normally have bactericidal activity [1].

Anti-enterococcal beta-lactams are preferred for ampicillin-susceptible enterococci (ASE) infections. Aminopenicillins, such as ampicillin, amoxicillin and piperacillin, are more active than imipenem and penicillin. Vancomycin is recommended among patients with beta-lactam-resistant enterococci infection and those in whom the use of beta- lactams is limited because of adverse drug reactions. Linezolid and daptomycin are reserved for vancomycin-resistant enterococci [1]. Most *E. faecalis* infections are sensitive to ampicillin, but *E. faecium* infection is often ampicillin-resistant [2,3].

The reported mortality rate of enterococcal bloodstream infections (BSI) was approximately 23 to 50% [3,4,5,6,7,8,9,10,11]. Ampicillin or vancomycin resistance in enterococcal infections is a factor that had been associated with mortality [4,12]. The 28-day mortality rate was shown to be significantly lower in ASE bacteremia than in ampicillin-resistant enterococci (ARE) bacteremia [13]. The use of inappropriate antibiotics in enterococcal BSI has increased the risk of death [4,5]. In cases of ampicillin-susceptible *E. faecalis* bacteremia treated with appropriate antibiotics, the mortality rate was higher with glycopeptides than with beta-lactams [9]. However, few studies focused on vancomycin increasing mortality among patients with ASE BSI. The other reported risk factors associated with mortality from enterococcal bacteremia were the type of enterococcal species, the severity of infections, and comorbidities [3,6,9,10].

Beta-lactams do not exhibit bactericidal activity to enterococci. For enterococcal endocarditis, beta-lactam monotherapy has a high relapse rate; therefore, the addition of aminoglycosides to beta-lactams is recommended for synergistic bactericidal activity to treat ASE endocarditis. Dual beta-lactam regimens, e.g., ampicillin with ceftriaxone, are recommended only for ampicillin-susceptible *E. faecalis* endocarditis and are often used in cases with high-level gentamicin/streptomycin resistance or risk of kidney toxicity [14]. The role of combination therapy for other infection sites remains unclear [8,15]. Some infectious disease experts recommended combination therapy for enterococcal BSI [16].

There are few studies on the outcomes and factors related to mortality among patients with ASE BSI. In the present study, we aimed to determine the treatment outcomes, characteristic and treatment-related factors on the 30-day mortality and 30-day survival time of patients with ASE bacteremia.

## 2. Materials and Methods

### 2.1. Study Design and Patient Populations

This retrospective chart review study was performed on patients with ASE bacteremia and admitted between 1 January 2013 and 30 April 2022 at Phramongkutklao Hospital, which is a 1200-bed medical school hospital in Bangkok, Thailand. Patients aged ≥ 20 years, with ASE identified in blood and receiving appropriate anti-enterococcal antibiotics for three days were included. Patients who were unable to follow-up to determine treatment outcome were excluded from this study.

### 2.2. Ethics Approval, Data Collection and Outcomes

The study was approved by the Institutional Review Board of the Royal Thai Army Medical Department at Phramongkutklao College of Medicine and Phramongkutklao Hospital (Approval number Q020h/65). Data were collected after ethics approval and permission from the Director of Phramongkutklao Hospital. The patient data obtained from the electronic medical records included (1) demographic data, such as sex, age, immune status and age-adjusted Charlson Comorbidity Index (ACCI) [17]; (2) infection data, such as days from admission to bacteremia onset, site of infection, species of enterococci, susceptibility result, co-infecting organisms, antibiotic treatment for ASE bacteremia, days until appropriate antibiotic treatment and source control; (3) severity of illness, based on admitting ward, septic shock, mechanical ventilation use and Pitt bacteremia score (PBS) at onset of bacteremia; (4) clinical outcomes, including 30-day all-cause mortality, in-hospital all-cause mortality, time to clinical stability, relapse infection and length of stay; (5) microbiological outcomes (i.e., microbiological clearance) and (6) adverse outcomes, such as acute kidney injury (AKI) and vancomycin-resistant enterococci (VRE) isolation.

### 2.3. Definitions

ASE bacteremia was defined as isolation of enterococci in at least one blood culture. Septic shock was defined according to the Third International Consensus Definitions for sepsis and septic shock [18]. Immunocompromised patients were defined as those who had organ transplant, human immunodeficiency virus infection with CD4 count < 100 cells/mm^3^, neutropenia (neutrophil count < 500/mm^3^), or those receiving immunosuppressive drugs within 30 days before ASE bacteremia. Hospital acquired bacteremia was defined as isolation of ASE from blood at least 48 h after hospital admission or within 48 h after hospital discharge. Polymicrobial bacteremia was defined as isolation of other pathogens from blood within 48 h of the positive blood culture for ASE. Polymicrobial infection was defined as the presence of other organisms on the same site of ASE isolation within 48 h. Anti-enterococcal antibiotics were defined as those used to treat enterococcal infection, including anti-enterococcal beta-lactams (penicillin, ampicillin, piperacillin/tazobactam and imipenem), vancomycin and linezolid. Appropriate treatment was defined as the administration of at least one active anti-enterococcal antibiotic, according to susceptibility testing, and the administration of an appropriate dose, according to vancomycin dosing guidelines [19]. Source control included removal of the invasive device, surgery, debridement, incision and drainage. Clinical stability was defined as achieving normal systolic blood pressure (≥100 mmHg), temperature (36 to 38 °C), heart rate (≤100 beats/min) and respiratory rate (<22 breaths/min) after appropriate treatment [20]. Length of stay was defined as length of time that elapsed between ASE BSI and discharge/death. Microbiological clearance was defined as negative blood culture after appropriate treatment. Relapse ASE bacteremia was defined as isolation of the same ASE isolate from blood within three months after completing treatment. According to the *2012 Kidney Disease: Improving Global Outcomes Clinical Practice Guideline*, the definition of AKI is an increase in serum creatinine by ≥0.3 mg/dL within 48 h, an increase in SCr to ≥1.5 times from baseline within the preceding seven days, or a urine volume of <0.5 mL/kg/h for six hours [21]. VRE isolation was defined as isolation of enterococci that are resistant to vancomycin from any specimen within three months after ASE bacteremia treatment. 

### 2.4. Statistical Analysis

The statistical software SPSS for Windows Version 27 (IBM Corp., Armonk, NY, USA) was used for analysis. The patient and infection characteristics were analysed with descriptive statistics. Continuous data were compared using the Student’s t-test or Mann–Whitney U test. Pearson’s chi-square test or Fisher’s exact test was used to compare categorical variables. A logistic regression analysis was used to determine the factors associated with 30-day mortality. Variables with a *p* value of ≤0.1 in the univariate analysis were included for backward stepwise multivariate analysis. For 30-day survival, variables that were significant on univariate analysis were incorporated in Cox’s regression analysis. A *p* value of ≤0.05 was considered statistically significant. 

## 3. Results

During the study period, 187 patients experienced 202 episodes of positive blood cultures for ASE. Thirty-seven patients did not receive treatment because of transient bacteremia (*n* = 28), palliative care (*n* = 9) and receipt of inappropriate treatment (*n* = 2). Three patients were referred to another hospital. Therefore, 145 patients with ASE BSI were included in this study. 

### 3.1. Patient and Infection Characteristics

In total, the median age was 75 years (IQR, 62 to 81 years) and the median ACCI was six points (IQR, 4 to 7 points). Ninety-one (56.9%) patients acquired infection in the hospital, 58 (36.3%) were placed on mechanical ventilation, and 37 (23.1%) presented septic shock (Table 1). *E. faecalis* was the most common strain (86.3%). *E. faecium* and other species were 9.3 and 4.3%, respectively. One was identified with both AS-*E. faecalis* and AS-*E. faecium*. *Enterobacteriaceae* (*n* = 33), *Staphylococcus* (*n* = 6), *Pseudomonas aeruginosa* (*n* = 5) and *Candida albicans* (*n* = 1) were found among patients with polymicrobial bacteremia. Anti-enterococcal beta-lactams monotherapy was most commonly prescribed for ASE BSI. Vancomycin was not used to treat ASE infective endocarditis (Table S1).

### 3.2. Treatment Outcomes

The 30-day mortality totaled 46 (28.8%) and the in-hospital mortality was 67 (41.9%). The median in-hospital survival time of patients with ASE BSI was 20 days (95% CI 14.27 to 25.73). Of 105 patients having microbiological data after treatment, 76 (73.1%) achieved microbiological cure (Table 1). Compared with survivors, patients who died within 30 days had higher ACCI and PBS (Table 2). The 30-day mortality among patients receiving vancomycin was 61.5%, compared with 26% among those receiving beta-lactam monotherapy (OR 4.55, 95% CI 1.39 to 14.92) and 25% among those receiving a beta-lactam combination (OR 4.8, 95% CI 1.13 to 20.46) (Table 3). Compared with the other groups, the vancomycin treatment group presented less stable clinical signs (*p* < 0.001) (Table S1). Within three months after ASE bacteremia treatment, 5 (3.1%) patients had vancomycin-resistant enterococci identified in various specimens (Table 1).

### 3.3. Factors Associated with All-Cause 30-Day Mortality and 30-Day Survival Time

**Figure 1:**
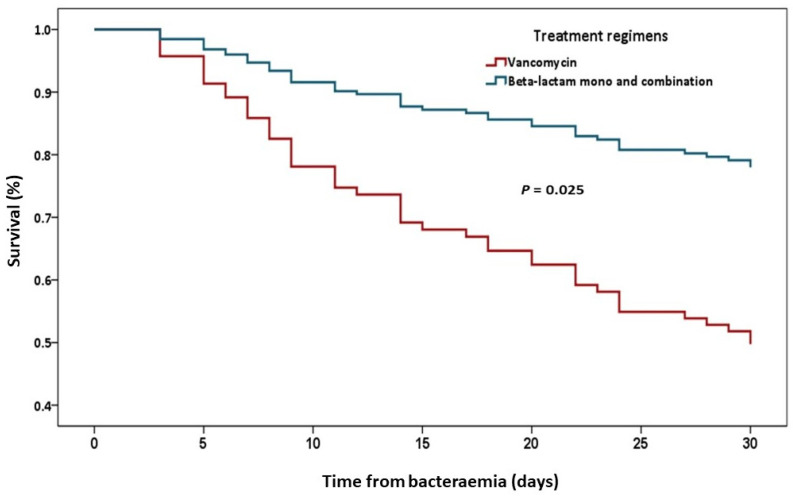
Kaplan-Meier survival curve for 30-day mortality between antibiotic regimens.

On univariate analysis, the factors related to 30-day mortality were age, ACCI, PBS, bacteremia onset, admission to the intensive care unit, use of mechanical ventilation, septic shock, receipt of vancomycin monotherapy and not performing the source control process. Concerning multivariate logistic regression analysis, PBS (OR 1.44, 95% CI 1.20 to 1.71); ACCI (OR 1.34, 95% CI 1.14 to 1.58) and receipt of vancomycin (OR 4.07, 95% CI 1.02 to 16.22) were significant factors associated with 30-day mortality (Table 4). Regarding Cox-regression survival analysis, the independent factors related to 30-day mortality were ACCI, PBS and receipt of vancomycin monotherapy. The comparison of the Kaplan-Meier survival curves of 30-day mortality between vancomycin and the other treatment regimens is shown in Figure 1.

## 4. Discussion

This is one of the few studies concerning the mortality rate of ASE bacteremia. The 30-day mortality rate was high despite the low virulence of the pathogen [22]. Compared with related studies on ASE bacteremia or beta-lactam-susceptible *E. faecalis* bacteremia [9,13,23], the present study had patients with similar baseline characteristics and treatment but higher mortality. Our results were similar to studies on enterococcal BSI, regardless of ampicillin sensitivity [3,5]. Many of our patients were older; had more comorbidities, severe illness and polymicrobial infections. Patients who had severe illness were treated as having enterococcal infection by physicians. Unsurprisingly, we found that the severity of illness and comorbidities were independent factors related to mortality, consistent with related studies [4,7,9,10].

Compared with beta-lactams, vancomycin therapy increased mortality and was one of the independent factors associated with 30-day mortality. Compared with the beta-lactam group, the vancomycin treatment group had shorter time to appropriate therapy as empiric vancomycin for nosocomial infection without de-escalation. These results were comparable with those of the study by Foo H et al., who reported that glycopeptide therapy was one of the independent factors for 30-day mortality in beta-lactam and vancomycin-susceptible *E. faecalis* bacteremia [9]. In contrast, the study by Fletcher J et al., found that 30-day all-cause mortality did not differ between beta-lactam and vancomycin therapy, but one year mortality was higher with vancomycin than with beta-lactam [23]. This could be attributed to the monitoring of serum vancomycin trough levels in the study by Fletcher J et al. Inappropriate antibiotic therapy was associated with mortality [5]. The appropriate dose of vancomycin in our study was determined based on guideline recommendations, and was not adjusted according to serum vancomycin levels. Data on drug concentrations were unavailable and the timing of concentrations was not determined. The association between the pharmacokinetics and pharmacodynamic targets of vancomycin and the outcome of enterococcal infection was unclear. An area under the curve (AUC)/minimum inhibitory concentration (MIC) of <400 mg/L/h had been associated with clinical failure in ARE infection [24], and an AUC/MIC of <389 mg/L/h was associated 30-day mortality in enterococcal bacteremia [25]. Nakakura I’s study did not find any correlation [26]. The MIC breakpoint of vancomycin for enterococci is 4 µg/mL [27]; unfortunately, in our study, AUC/MIC in each patient could not be evaluated.

Among patients with methicillin-susceptible *Staphylococcus aureus* bacteremia, vancomycin was reported to increase mortality and was inferior to beta-lactams [28]. It exhibits a slow bactericidal activity against methicillin-susceptible *S. aureus* [29,30]. In an in vitro *E. faecalis* infected fibrin–platelet clot model, a decrease in bacterial load at 72 h was higher with ampicillin than with vancomycin with or without gentamicin; ampicillin was superior to vancomycin most likely because of its extent of antibiotic penetration through fibrin [31]. Time-kill studies on 15 *E. faecalis* strains found that the bactericidal kinetics of vancomycin and teicoplanin were slower and observed at earlier periods compared with those of beta-lactams [32].

Combined anti-enterococcal beta-lactam with gentamicin is commonly used for infective endocarditis and infections of an unknown source. In our study, combination therapy did not improve outcomes. A small number of infective endocarditis cases have been conducted, and some cases of endocarditis were not treated with combination therapy, although they were not highly resistant to gentamicin. However, this study was not designed to compare the efficacy of beta-lactam monotherapy to combination therapy. The benefits of combination therapy other than those for endocarditis remain unclear. Combination therapy had been recommended until infective endocarditis is ruled out [8,15,16,33]. A management bundle that includes echocardiography, blood culture follow-up and treatment with combination antibiotics for BSI of unknown source was reported to improve the 30-day survival of enterococcal BSI [34]. In our study, most of the relapse cases had unknown sources of infection and were diagnosed as endocarditis for a second bacteremia. However, the benefits and harms of anti-enterococcal beta-lactam combination therapy for non-endocarditis BSI should be weighed.

In our study, no cases of relapse bacteremia and death were found among patients who were switched to oral medications including amoxicillin and amoxicillin-clavulanic acid. Amoxicillin has moderate oral bioavailability [35]. Data on oral antibiotics to treat enterococcal BSI is limited. The oral beta-lactam step-down was non-inferior to fluoroquinolones to treat uncomplicated streptococcal BSI [36]. Therefore, switching to oral therapy to reduce the length of hospital stay and the risk of nosocomial infection may be effective in enterococcal BSI [37].

No differences were found in the incidence of AKI and VRE isolation between the antibiotic regimens. A small number of cases of VRE isolation after ASE treatment were noted. Several factors have been discovered for VRE isolation, and previous longer exposure to broad spectrum antibiotics was also a risk factor. Several antibiotics, including anti-anaerobic agents, fluoroquinolones and vancomycin, have been studied [38,39,40]. In our study, the low prevalence of VRE isolation may be attributed to the short follow-up period; approximately one half of cases were community-acquired and only some cases collected specimens for VRE isolation.

Several limitations were encountered in the current study. First, this comprised a retrospective study with a small sample size, making it difficult to determine other factors related to mortality. Moreover, outcomes were not collected in all cases. Second, the appropriate dose of vancomycin was not adjusted by vancomycin MIC and therapeutic drug monitoring. Further studies where serum monitor vancomycin and have a larger sample size are required to identify the outcomes of vancomycin use and treatment regimens among patients with ASE BSI.

## 5. Conclusions

The mortality rate of ASE BSI was high. The mortality rate and 30-day survival were associated with multiple comorbidities, more severe illness and definitive treatment with vancomycin. Anti-enterococcal beta-lactams remain the first line antibiotics. However, vancomycin might be an alternative treatment for those who have severe allergic reactions to beta-lactams. 

## Figures and Tables

**Table 1:** Characteristics and outcomes of patients with ampicillin-susceptible enterococci bloodstream infections.

Characteristic	Number (%)
Male	86 (59.3)
Age (years), median (IQR)	75 (62–81)
ACCI, median (IQR)	6 (4–7)
Immunocompromised status	23 (14.4)
Time from admission to bacteremia onset (days), median (IQR)	4 (0–18.75)
Hospital acquired bacteremia	91 (56.9)
Intensive-care unit	35 (21.9)
Mechanical ventilation	58 (36.3)
Septic shock	37 (23.1)
PBS, median (IQR)	2 (0–4)
Sites of infection	
Primary bacteremia	47 (29.4)
Intraabdominal infection	35 (21.9)
Intravascular catheter infection	26 (16.3)
Urinary system infection	21 (13.1)
Infective endocarditis	12 (7.5)
Skin and soft tissue infection	9 (5.6)
Bone and joint infection	6 (3.8)
Lower respiratory tract infection (Empyema thoracis)	3 (1.9)
Reproductive tract infection	1 (0.6)
Polymicrobial bacteremia	41 (25.6)
Polymicrobial infection	49 (30.6)
Time to appropriate therapy (days), median (IQR)	1 (0–3)
Definitive antibiotic regimens	
Anti-enterococcal beta-lactams	123 (76.9)
Vancomycin	13 (8.1)
Anti-enterococcal beta-lactams—Gentamicin	14 (8.8)
Anti-enterococcal beta-lactams—Ceftriaxone	3 (1.9)
Anti-enterococcal beta-lactams—Vancomycin	6 (3.8)
Anti-enterococcal beta-lactams—Tigecycline	1 (0.6)
Switched therapy from IV to oral	22 (13.8)
Source control	50 (31.3)
Treatment outcomes	
All-cause 30-day mortality	46 (28.8)
All-cause in hospital mortality	67 (41.9)
Clinical stability	114 (71.3)
Time to clinical stability (days); median (IQR)	3 (2–6)
Length of stay after infection (days); median (IQR)	19 (13–34)
Relapsed ASE bacteremia; median (IQR) = 29 (24–38) days	7 (4.4)
Microbiological clearance within 7 days (*n* = 104)	76 (73.1)
Adverse outcomes	
Vancomycin-resistant enterococci *	5 (3.1)
Acute kidney injury	26 (16.3)

* Colonization (*n* = 3), infection (*n* = 2). Abbreviations: ACCI, age-adjusted Charlson Comorbidity Index; IV, intravenous; IQR, interquartile range; PBS, Pitt bacteremia score.

**Table 2:** Comparison of the characteristics between survivors and no survivors within 30-days of ampicillin–susceptible enterococci bloodstream infections.

Characteristic	Death (*n* = 46)	Survival (*n* = 114)	*p* Value	OR	95% CI
Mal	25 (54.3)	71 (62.3)	0.354	0.72	0.36–1.44
Age (years)	77 (65–85)	74 (61–80)	0.052 *		
ACCI; median (IQR)	6.5 (5–8)	5 (4–7)	<0.001 *		
ACCI 0–2 (*n* = 21)	0	21			
ACCI 3–5 (*n* = 58)	15 (32.9)	43 (37.7)			
ACCI 6–13 (*n* = 81)	31 (67.4)	50 (43.9)			
Immunocompromised	9 (19.6)	14 (12.3)	0.235	1.74	0.69–4.35
Time from admission to bacteremia (days); median (IQR)	10 (2.5–31.5)	0 (0–12)	0.001 *		
HA-bacteremia	35 (76.1)	56 (49.1)	0.002	3.3	1.52–7.12
Intensive care unit	18 (39.1)	17 (14.9)	0.001	3.67	1.67–8.04
Mechanical ventilation	26 (56.5)	32 (28.1)	0.001	3.33	1.64–6.79
Septic shock	20 (43.5)	17 (14.9)	<0.001	4.39	2.02–9.56
PBS; median (IQR)	3.5 (2–5)	1 (0–3)	<0.001 *		
PBS 0–3 (*n* = 113)	22 (47.8)	91 (79.8)			
PBS 4–7 (*n* = 41)	20 (43.5)	21 (18.4)			
PBS 8–11 (*n*= 6)	4 (8.7)	2 (1.8)			
Site of infection					
Primary bacteremia	17 (37)	30 (26.3)	0.181	1.64	0.79–3.4
Intraabdominal infection	13 (28.3)	22 (19.3)	0.215	1.65	0.75–3.64
Intravascular catheter infection	6 (13)	20 (17.5)	0.485	0.71	0.26–1.89
Urinary system infection	6 (13)	15 (13.2)	0.985	1	0.36–2.73
Infective endocarditis	2 (4.3)	10 (8.8)	0.511	0.47	0.1–2.25
Skin and soft tissue infection	1 (2.2)	8 (7)	0.448	0.29	0.04–2.42
Bone and joint infection	0	6 (5.3)	0183	-	-
Lower respiratory tract infection (Empyema thoracis)	1 (2.2)	2 (1.8)	1.0	1.24	0.11–14.07
Reproductive tract infection	0	1 (0.9)	1.0	-	-
Species of enterococci					
*E. faecalis* (139)	41 (89.1)	97 (85.8)	0.591	1.34	0.46–3.9
*E. faecium* (15)	5 (10.9)	9 (8)	0.546	1.42	0.45–4.5
Other species (7)	0	7 (6.2)	0.194	-	-
Polymicrobial bacteremia	11 (23.9)	30 (26.3)	0.75	0.88	0.4–1.95
Polymicrobial infection	13 (28.3)	36 (31.6)	0.68	0.85	0.4–1.81
Time to appropriate therapy (days); median (IQR)	1 (0–2)	1 (0–3)	0.337 *		
Received appropriate therapy within 1 day	33 (71.7)	68 (60.2)	0.170	1.68	0.8–3.54
Definitive antibiotic regimens				0.024	
Anti-enterococcal beta-lactams	32 (69.6)	91 (79.8)	0.164	0.58	0.27–1.26
Anti-enterococcal beta-lactams combination	6 (13)	18 (15.8)	0.66	0.8	0.3–2.16
Vancomycin	8 (17.4)	5 (4.4)	0.011	4.59	1.42–14.89
Source control	8 (17.4)	42 (36.8)	0.016	0.36	0.15–0.85

* Mann–Whitney U test. Abbreviations: ACCI, age-adjusted Charlson Comorbidity Index; CI, confidence interval; HA—bacteremia, hospital acquired bacteremia; HLGR, high level gentamicin resistance; IQR, interquartile range; OR, odd ratio; PBS, Pitt bacteremia score.

**Table 3:** Thirty-day mortality rate between antibiotic regimens to treat patients with ampicillin susceptible enterococci bloodstream infections.

Regimens between Group 1 and Group 2 ^1^	*p* Value	OR	95%CI
Vancomycin vs. Anti-enterococcal beta-lactams	0.02	4.55	1.39–14.92
Vancomycin vs. Anti-enterococcal beta-lactams combination	0.039	4.8	1.13–20.46
Anti-enterococcal beta-lactams vs. Anti-enterococcal beta-lactams combination	0.917	1.06	0.38–2.89

^1^ Group 2 as reference. Abbreviations: CI, confidence interval; OR, odd ratio.

**Table 4:** Factors associated with all-cause 30-day mortality on multivariate logistic regression analyses.

Variable	*p* Value	OR	95% CI
Age (years)	0.963	1.0	0.96–1.04
Time from admission to bacteremia (days)	0.628	0.99	0.98–1.01
HA-bacteremia	0.48	0.69	0.25–1.91
ACCI (each increase of 1 point)	<0.001	1.34	1.14–1.58
PBS (each increase of 1 point)	<0.001	1.44	1.20–1.71
Intensive-care unit	0.154	2.28	0.73–7.12
Mechanical ventilation	0.575	0.67	0.17–2.7
Septic shock	0.403	1.72	0.48–6.21
Vancomycin monotherapy	0.047	4.07	1.02–16.22
Source control	0.135	0.44	0.15–1.29

Abbreviations: ACCI, age-adjusted Charlson Comorbidity Index; CI, confidence interval; OR, odd ratio; PBS, Pitt bacteremia score.

## Data Availability

The data used to support the finding of this study are included within the article.

## Supplementary Materials

The following supporting information can be downloaded at: https://www.mdpi.com/article/10.3390/antibiotics11121698/s1, Table S1: Comparing characteristics and outcomes of beta-lactams, beta-lactams combination and vancomycin among patients with ampicillin-susceptible enterococci bloodstream infections.
