# Supplementary material for: Clinical Impact of Vancomycin Treatment in Ampicillin-Susceptible Enterococci Bloodstream Infections
Source: Antibiotics (Basel). 2022 Nov 25;11(12):1698. doi: 10.3390/antibiotics11121698 (PMC9774542; doi:10.3390/antibiotics11121698)
Supplement: Supplementary file 1 [file antibiotics-11-01698-s001.zip › antibiotics-2015502-supplementary.pdf]

**Table S1.** Comparing characteristics and outcomes of beta-lactams, beta-lactams combination and vancomycin among patients with ampicillin – susceptible enterococci bloodstream infections.

| Characteristics | Anti-enterococcal beta-lactams (*n*=123) | Anti-enterococcal beta-lactams combination (*n*=24) | Vancomycin (*n*=13) | *p* value |
|---|---|---|---|---|
| Male | 76 (61.8) | 14 (10) | 6 (46.2) | 0.557 |
| Age (years), median (IQR) | 75 (20) | 74 (16) | 71 (22) | 0.672 |
| ACCI, median (IQR) | 5 (3) | 6 (3) | 6 (2) | 0.787 |
| Immunocompromised status | 19 (15.4) | 2 (8.3) | 2 (15.4) | 0.719 |
| Time from admission to bacteremia onset (days), median (IQR) | 4 (17) | 0 (19) | 18 (14) | 0.169 |
| Hospital acquired bacteremia | 68 (55.3) | 12 (50) | 11 (84.6) | 0.089 |
| Intensive – care unit | 26 (21.1) | 4 (16.7) | 5 (38.5) | 0.285 |
| Mechanical ventilation | 42 (34.1) | 8 (33.3) | 8 (61.5) | 0.155 |
| Septic shock | 27 (22.0) | 6 (25.0) | 4 (30.8) | 0.752 |
| PBS, median (IQR) | 2 (4) | 1.5 (4) | 3 (5) | 0.242 |
| Sites of infection | | | | |
| Primary bacteremia | 35 (28.5) | 7 (29.2) | 5 (38.5) | 0.753 |
| Intraabdominal infection | 28 (22.8) | 3 (12.5) | 4 (30.8) | 0.388 |
| Intravascular catheter infection | 18 (14.6) | 5 (20.8) | 3 (23.1) | 0.508 |
| Urinary system infection | 19 (15.4) | 1 (4.2) | 1 (7.7) | 0.32 |
| Infective endocarditis | 5 (4.1) | 7 (29.2) | 0 | 0.001 * |
| Skin and soft tissue infection | 9 (7.3) | 0 | 0 | 0.368 |
| Bone and joint infection | 5 (4.1) | 1 (4.2) | 0 | 1.0 |
| Lower respiratory tract infection (Empyema thoracis) | 3 (2.4) | 0 | 0 | 1.0 |
| Reproductive tract infection | 1 (0.8) | 0 | 0 | 1.0 |
| Species | | | | |
| *E. faecalis* | 107(87) | 23 (95.8) | 9 (69.2) | 0.08 |
| *E. faecium* | 10 (8.1) | 0 | 4 (30.8) | 0.011 * |
| *E. gallinarum* | 2 (1.6) | 0 | 0 | 1.0 |
| *E. hirae* | 1 (0.8) | 0 | 0 | 1.0 |
| *Enterococcus* spp. | 3 (2.4) | 1 (4.2) | 0 | 1.0 |
| Polymicrobial bacteremia | 34 (27.6) | 5 (20.8) | 2 (15.4) | 0.531 |
| Polymicrobial infection | 41 (33.3) | 5 (20.8) | 3 (23.1) | 0.395 |
| Time to appropriate therapy (days), median (IQR) | 1 (3) | 0 (2) | 0 (1) | 0.021 |
| Switch therapy from IV to oral | 20 (16.3) | 2 (8.3) | 0 | 0.275 |
| Source control | 39 (31.7) | 8 (33.3) | 3 (23.1) | 0.793 |
| Treatment outcomes | | | | |
| All-cause 30 – day mortality | 32 (26.0) | 6 (25.0) | 8 (61.5) | 0.024 * |
| All-cause in – hospital mortality | 50 (40.7) | 8 (33.3) | 9 (69.2) | 0.092 |
| Clinical stability | 94 (76.4) | 17 (70.8) | 3 (23.1) | <0.001 * |

| Characteristics | Anti-enterococcal beta-lactams (*n*=123) | Anti-enterococcal beta-lactams combination (*n*=24) | Vancomycin (*n*=13) | *p* value |
|---|---|---|---|---|
| Time to clinical stability (days); median (IQR) | 4 (5) | 5 (7) | 3 (-*) | 0.494 |
| Length of stay after infection (days); median (IQR) | 18 (22) | 22 (31) | 15 (17) | 0.184 |
| Relapse ASE bacteremia; median (IQR) | 6 (4.9) | 1 (4.2) | 0 | 1.0 |
| Microbiological clearance within 7 days (N=104) | 64 (52) | 11 (45.8) | 9 (69.2) | 0.379 |
| **Adverse outcomes** | | | | |
| Vancomycin – resistant enterococci* | 3 (2.4) | 1 (4.2) | 1 (7.7) | 0.327 |
| Acute kidney injury | 18 (14.6) | 6 (25.0) | 2 (15.4) | 0.439 |

**Abbreviations:** ACCI, age-adjusted Charlson Comorbidity Index; IV, intravenous; IQR, interquartile range; PBS, Pitt bacteremia score

* The 25th percentile: 2, the 50th percentile: -
